# Gestational diabetes mellitus and risk of long-term all-cause and cardiac mortality: a prospective cohort study

**DOI:** 10.1186/s12933-024-02131-3

**Published:** 2024-02-01

**Authors:** Qian Ying, Yao Xu, Ziyi Zhang, Luyi Cai, Yan Zhao, Liping Jin

**Affiliations:** 1 Shanghai Key Laboratory of Maternal Fetal Medicine, Shanghai First Maternity and Infant Hospital, School of Medicine, Tongji University, Shanghai, China; 2 Shanghai Key Laboratory of Female Reproductive Endocrine Related Diseases, Hospital of Obstetrics and Gynecology, Shanghai Medical School, Fudan University, Shanghai, China

**Keywords:** Gestational diabetes mellitus, Overt diabetes, All-cause mortality, Cardiac mortality, Hazard ratio, Cohort study

## Abstract

**Background:**

To investigate the association between gestational diabetes mellitus (GDM) without subsequent overt diabetes and long-term all-cause and cardiac mortality.

**Methods:**

This prospective cohort study included 10,327 women (weighted population: 132,332,187) with a pregnancy history from the National Health and Nutrition Examination Survey (2007 to 2018). Participants were divided into three groups (GDM alone, overt diabetes, and no diabetes). Mortality data was linked from the National Death Index up to December 31, 2019. Multivariable Cox regression analysis was performed to examine the association between GDM alone and overt diabetes with all-cause mortality and cardiac mortality. Data analysis was performed from October 2022 to April 2023.

**Results:**

Among the participants, 510 (weighted 5.3%) had GDM alone and 1862 (weighted 14.1%) had overt diabetes. Over a median follow-up period of 6.7 years (69,063 person-years), there were 758 deaths. The GDM group did not show an increased risk of all-cause mortality (hazard ratio [HR] 0.67; 95% CI, 0.25–1.84), while the overt diabetes group had a significantly higher risk (HR 1.95; 95% CI, 1.62–2.35). Similarly, the GDM group did not exhibit an elevated risk of cardiac mortality (HR 1.48; 95% CI, 0.50–4.39), whereas the overt diabetes group had a significantly higher risk (HR 2.37; 95% CI, 1.69–3.32). Furthermore, sensitivity analysis focusing on women aged 50 or above showed that the HR of GDM history for all-cause mortality was 1.14 (95% CI, 0.33–3.95) and the HR for cardiac mortality was 1.74 (95% CI, 0.49–6.20).

**Conclusions:**

GDM alone was not associated with an increased risk of all-cause and cardiac mortality, while overt diabetes was significantly associated with both types of mortality.

## Background

Gestational diabetes mellitus (GDM) refers to abnormal glucose intolerance diagnosed for the first time during pregnancy. It is considered one of the frequent complications during gestation, with a global prevalence of around 14% [1]. In the United States, the incidence of GDM is also on the rise [2]. While most women regain normal glucose tolerance within a few days after delivery, extensive studies have indicated that GDM not only impacts short-term pregnancy outcomes but also possesses long-lasting health implications, particularly in the development of cardiac diseases [3–5]. Additionally, it is worth noting that women who have had GDM are at an approximately 10-fold higher risk of developing type 2 diabetes than those with normal blood glucose levels during pregnancy [6]. The progression to overt diabetes further compounds the long-term impact on health [7]. However, the specific impact of a history of GDM alone on overall and cause-specific mortality in women has not been widely studied. In order to fill this gap, we conducted an analysis using data from National Health and Nutrition Examination Survey (NHANES) linked with the National Death Index (NDI) data to explore the correlation between a history of GDM without subsequent progression to overt diabetes and overall mortality, as well as cardiac mortality, in women.

## Methods

This prospective cohort study utilized a nationally representative sample from the NHANES database of the United States. NHANES conducts surveys every two years beginning in 1999, wherein participants are invited to complete an in-person interview and undergo a series of physical and laboratory examinations at a mobile examination center to collect information on health and nutrition. All NHANES protocols were approved by the National Center for Health Statistics ethics review board, and written informed consent has been obtained from all participating individuals.

This research included women with a history of pregnancy from the NHANES data spanning six cycles from 2007 to 2018. Participants with missing information regarding GDM and overt diabetes as well as those potential confounding factors were excluded.

### Diagnosis of GDM and overt diabetes

Information about GDM was gathered during the in-person interview through the reproductive questionnaire (RHQ162). Participants were asked, “During pregnancy, were you ever told by a doctor or other health professional that you had diabetes, sugar diabetes, or gestational diabetes? (do not include diabetes that you may have known about before the pregnancy)”. Individuals who answered “yes” were defined as having GDM.

The identification of overt diabetes was divided into two parts. Firstly, during the in-person interview of the diabetes questionnaire (DIQ10), participants were asked, “Other than during pregnancy, have you ever been told by a doctor or health professional that you have diabetes or sugar diabetes?“. Individuals who answered “yes” were defined as having diagnosed diabetes. To prevent missed cases of undiagnosed diabetes, we also examined the laboratory test results of participants. Individuals who exhibited an HbA1c level greater than 6.5%, fasting plasma glucose (FPG) level greater than 126 mg/dL (7.0 mmol/L), or 2-hour postprandial glucose (2hPG) level greater than 200 mg/dL (11.1 mmol/L) were categorized as having undiagnosed diabetes. Total diabetes was defined as self-reported diagnosed diabetes or undiagnosed diabetes.

### Ascertainment of mortality

The NDI is a comprehensive computerized database of death records in the United States, consisting of records submitted by the Vital Statistics Office of each state to the National Center for Health Statistics since 1979. Reporting to the NDI is mandatory and encompasses the entire U.S. population. The primary source of data within the NDI is state-mandated death certificates. These death records are collected at the local level, compiled at the country and state levels, and then transmitted to the NCHS Division of Vital Statistics by each state’s vital statistics office. Updates to the NDI file occur annually, about 11 months after the end of the calendar year, incorporating decedent information provided by state vital records offices to the NCHS. Rigorous procedures, including meticulous verification, quality control measures, and regular updates, are implemented to ensure the reliability of the data. Previous studies have confirmed the ability of the NDI to match decedents [8–10]. We utilized death data from the NDI up to December 31, 2019 (https://www.cdc.gov/nchs/data-linkage/mortality-public.htm), and linked it with NHANES data according to official methodology [11]. In this study, the cause of death was recorded using the International Statistical Classification of Diseases and Related Health Problems, Tenth Revision (ICD-10). Cardiac mortality was defined as death caused by heart (ICD-10 codes I00-I09, I11, I13, I20-I51). Person-years were calculated from the baseline, which was the time of participation in the NHANES survey, until the date of death or 31 December 2019, whichever came first. Data analysis was conducted between October 2022 and April 2023.

### Sociodemographic characteristics, lifestyle behaviors, and metabolic disease conditions

The study collected self-reported sociodemographic characteristics, including age, race and ethnicity, education level (categorized as < high school, high school or some college, ≥4y of college), and family poverty income ratio (calculated as total family income divided by the poverty threshold and categorized as < 1.3, 1.3 to 3.49, ≥ 3.5). Participants’ weight and height were measured during the physical examination and body mass index (BMI) was calculated (categorized as < 18.5, 18.5–24.9, 25-29.9, 30-34.9, ≥ 35). Lifestyle factors were also assessed, including smoking status (categorized as now, past, never), alcohol consumption (heavy drinker defined as ≥ 1 drink/day, low to moderate drinker defined as < 1 drink a day, and non-drinker), and physical activity (categorized as inactive, insufficiently active, active). Physical activity was determined based on the total amount of leisure-time physical activity (LTPA), which was estimated as the sum of minutes of moderate intensity recreational activities plus twice the minutes of vigorous-intensity recreational activities [12]. Participants were classified as inactive, insufficiently active, or sufficiently active based on their LTPA levels during the previous week, with LTPA of 0 min per week, less than 150 min/wk, and 150 min/wk or more, respectively.

The number of pregnancies was collected based on the in-person interview of the reproductive questionnaire (RHQ160). Hypertension was determined either through self-reporting by participants who had received a diagnosis from a health professional or through measurement of blood pressure during the NHANES survey (defined as ≥ 130 mm Hg [systolic] or ≥ 80 mmHg [diastolic]). Hypercholesterolemia was determined either through self-reporting by participants who had received a diagnosis from a health professional or through measurement of total cholesterol level during the NHANES survey (defined as ≥ 240 mg/dL). A history of cardiac disease (CVD) and/or cancer was self-reported by participants who had received either or both of these diagnoses from a health professional.

### Statistical analysis

The study followed the NHANES analytic guidelines to ensure nationally representative estimates. The analysis accounted for the unequal probability of selection, oversampling of certain subpopulations, and nonresponse adjustments to address potential biases.

We used the Rao-Scott χ2 test to compare the differences in baseline characteristics among participants. The participants were divided into three groups based on their GDM and diabetes status: no diabetes, GDM alone, and overt diabetes. Multivariable Cox proportional hazards regression models were utilized to estimate hazard ratios (HR) and 95% confidence intervals (CI) for the associations of GDM alone with all-cause and cardiac mortality, respectively. Three models were employed for the analysis. Firstly, only age was adjusted for. Secondly, adjustments were made for race and ethnicity, education, family poverty income ratio, BMI, smoking status, alcohol consumption, and physical activity. Lastly, further adjustments were made for the number of pregnancies, hypertension, hypercholesterolemia, and history of cardiovascular disease or cancer. Finally, we conducted a sensitivity analysis to test the robustness of the results. We included women aged over 50 years old and ran complete case analysis. We chose 50 years old as the cutoff primarily because menopause typically occurs around this age, in order to avoid including individuals in the non-GDM group who had not experienced GDM at the time of participating in the NHANES survey but subsequently developed GDM during subsequent pregnancies.

We conducted 2-sided statistical test and considered statistical significance at a threshold of *P* < 0.05. Data analyses were performed using R studio, version 2023.06.2 + 561 (Posit Software, PBC).

## Results

### Baseline characteristics and follow-up period

**Fig. 1:**
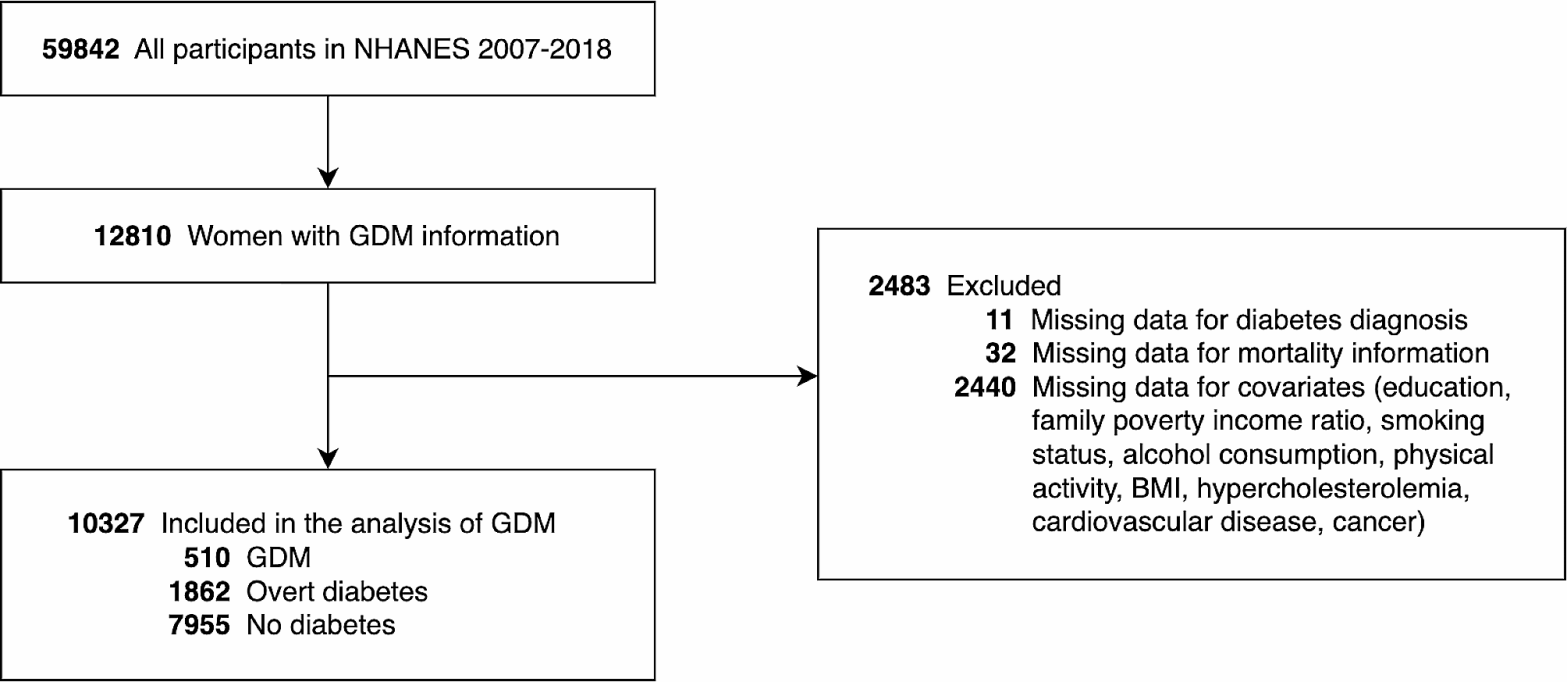
Flowchart of NHANES participants for the current analysis. Abbreviations: GDM, gestational diabetes mellitus

The study cohort consisted of 10,327 female participants (weighted population: 132,332,187) (Fig. 1). Among them, 7955 (weighted population: 106,690,725, 80.6%) had no diabetes, 510 (weighted population: 7,032,609, 5.3%) had a history of GDM alone, and 1862 (weighted population: 18,608,853, 14.1%) had overt diabetes. The overt diabetes group had the highest median age, while the GDM group was relatively younger. Details of other confounding factors are presented in Table 1. The median follow-up time for this study was 6.7 years, with a maximum follow-up of 13.3 years, resulting in a total of 69,063 person-years. During the follow-up period, there were a total of 758 deaths (weighted all-cause mortality rate: 5.7%). Among them, 183 deaths were due to heart disease (weighted cardiac mortality rate: 1.3%).

**Table 1:** Sample size ^a^ and characteristics for diabetes status with survival among us women with pregnancy history, NHANES 2007 to 2018

Characteristics	No. of participants by diabetes status (weighted %)				
	All	No diabetes	GDM	Overt diabetes	*p*-value ^b^
Overall	10,327	7955	510	1862	
Weighted	132,332,187	106,690,725	7,032,609	18,608,853	
**Age group, y**					< 0.001
< 30	1116(11.4%)	1028 (13.0%)	58 (11.4%)	30 (2.0%)	
30–39	1744 (17.7%)	1474 (18.9%)	168 (31.1%)	102 (6.1%)	
40–49	1987 (19.9%)	1579 (20.2%)	169 (32.5%)	239 (13.5%)	
50–59	1765 (20.3%)	1318 (19.9%)	73 (16.0%)	374 (24.3%)	
60–69	1911 (15.9%)	1310 (15.0%)	31 (7.2%)	570 (24.6%)	
70–79	1133 (9.4%)	769 (8.4%)	7 (1.1%)	357 (18.5%)	
≥ 80	671 (5.3%)	477 (4.6%)	4 (0.7%)	190 (11.1%)	
**Race and ethnicity**					< 0.001
Non-Hispanic White	4253 (67.5%)	3421 (68.6%)	204 (65.7%)	628 (61.8%)	
Hispanic	2713 (13.1%)	2003 (12.7%)	146 (14.6%)	564 (15.4%)	
Non-Hispanic Black	2276 (12.5%)	1698 (12.0%)	83 (9.5%)	495 (16.3%)	
Other	1085 (6.9%)	833 (6.7%)	77 (10.2%)	175 (6.5%)	
**Education**					< 0.001
<High school	2460 (15.3%)	1723 (13.8%)	107 (17.1%)	630 (23.3%)	
High school or some college	5661 (56.5%)	4405 (56.2%)	272 (48.2%)	984 (61.0%)	
≥4y of college	2206 (28.2%)	1827 (29.9%)	131 (34.7%)	248 (15.7%)	
**Family poverty income ratio**					< 0.001
< 1.30	3476 (23.2%)	2572 (22.4%)	168 (21.8%)	736 (27.9%)	
1.30–3.49	3909 (36.5%)	2967 (35.1%)	193 (39.9%)	749 (42.8%)	
≥ 3.50	2942 (40.4%)	2416 (42.5%)	149 (38.3%)	377 (29.3%)	
**Smoking status**					< 0.001
Now	1794 (17.8%)	1444 (18.4%)	107 (20.1%)	243 (13.2%)	
Past	1962 (21.4%)	1430 (20.7%)	82 (18.6%)	450 (26.8%)	
Never	6571 (60.8%)	5081 (60.9%)	321 (61.3%)	1169 (60.0%)	
**Alcohol consumption**					< 0.001
Heavy drinker	616 (7.6%)	525 (8.1%)	25 (7.1%)	66 (4.9%)	
Low to moderate drinker	5282 (58.5%)	4284 (60.9%)	286 (59.1%)	712 (44.6%)	
Non-drinker	4429 (33.9%)	3146 (31.0%)	199 (33.8%)	1084 (50.5%)	
**Physical activity**					< 0.001
Active	2827 (31.9%)	2372 (34.1%)	156 (33.9%)	299 (18.7%)	
Insufficiently active	1680 (17.9%)	1299 (18.2%)	86 (16.3%)	295 (16.9%)	
None	5820 (50.2%)	4284 (47.7%)	268 (49.8%)	1268 (64.4%)	
**BMI (kg/m**^**2**^)					< 0.001
< 18.5	152 (1.7%)	136 (1.9%)	6 (1.3%)	10 (0.7%)	
18.5–24.9	2723 (29.1%)	2379 (32.6%)	115 (24.4%)	229 (10.7%)	
25-29.9	2993 (29.1%)	2395 (30.1%)	152 (29.3%)	446 (23.7%)	
30-34.9	2199 (19.9%)	1586 (18.4%)	120 (24.6%)	493 (26.5%)	
≥ 35	2260 (20.2%)	1459 (17.0%)	117 (20.4%)	684 (38.4%)	
**No. of pregnancies**					< 0.001
1	1412 (16.4%)	1183 (17.9%)	51 (10.2%)	178 (9.9%)	
2	2516 (27.4%)	2000 (27.6%)	122 (28.1%)	394 (26.0%)	
3	2405 (23.5%)	1906 (23.8%)	114 (22.8%)	385 (22.3%)	
4	1675 (15.1%)	1249 (14.2%)	97 (18.0%)	329 (18.6%)	
≥ 5	2319 (17.6%)	1617 (16.4%)	126 (20.8%)	576 (23.3%)	
**Hypertension**					< 0.001
No	4699 (51%)	4041 (55.2%)	316 (67.8%)	342 (20.1%)	
Yes	5628 (49.0%)	3914 (44.8%)	194 (32.2%)	1520 (79.9%)	
**Hypercholesterolemia**					< 0.001
No	6105 (59.6%)	5036 (63.1%)	366 (71.3%)	703 (35.2%)	
Yes	4222 (40.4%)	2919 (36.9%)	144 (28.7%)	1159 (64.8%)	
**Cardiovascular disease**					< 0.001
No	9436 (92.7%)	7452 (94.6%)	489 (96.3%)	1495 (80.4%)	
Yes	891 (7.3%)	503 (5.4%)	21 (3.7%)	367 (19.6%)	
**Cancer**					< 0.001
No	9203 (87.8%)	7138 (88.5%)	470 (89.2%)	1595 (83.3%)	
Yes	1124 (12.2%)	817 (11.5%)	40 (10.8%)	267 (16.7%)	
**Family history of diabetes** ^**c**^	4661 (42.1%)	3142 (37.2%)	290 (55.3%)	1229 (65.5%)	< 0.001

Abbreviations: GDM, gestational diabetes mellitus; BMI, body mass index

^a^ Weighted to be nationally representative

^b^ Chi-squared test with Rao & Scott’s second-order correction

^c^166 missing for family history of diabetes

## All-cause mortality and cardiac mortality

**Fig. 2:**
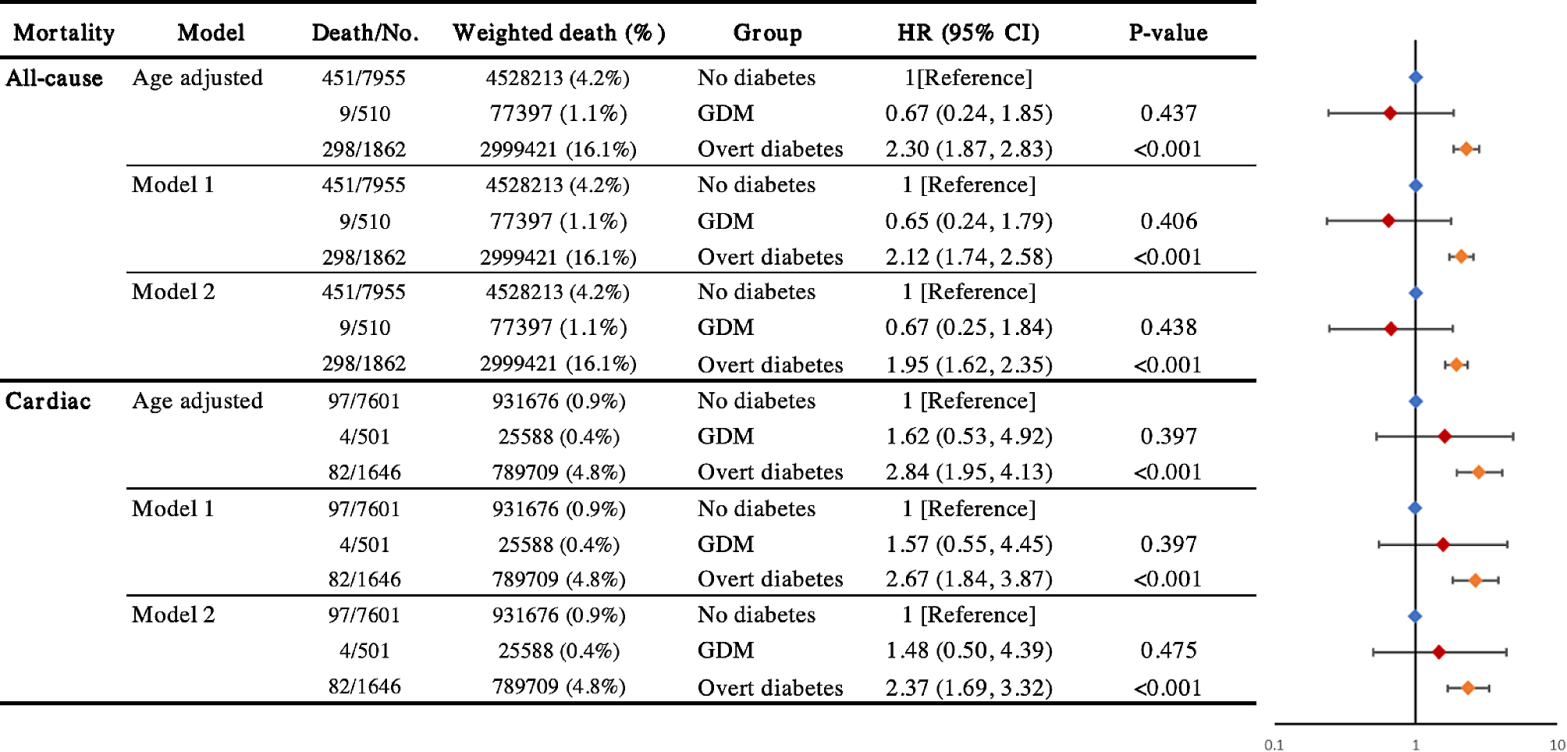
GDM With All-cause, Cardiac Mortality Among Us Women With Pregnancy History, NHANES 2007 to 2018. Model 1 used multivariable model adjusted for age, race and ethnicity, education, family poverty income ratio, BMI, smoking status, alcohol consumption, and physical activity. Model 2 additionally adjusted for no. of pregnancies, hypertension, hypercholesterolemia, history of cardiovascular disease or cancer

As shown in Fig. 2, over the follow-up period, the no diabetes group had 451 deaths (weighted mortality rate: 4.2%), the GDM group had 9 deaths (weighted mortality rate: 1.1%, HR 0.67; 95% CI, 0.25–1.84), and the overt diabetes group had 298 deaths (weighted mortality rate: 16.1%, HR 1.95; 95% CI, 1.62–2.35). During the follow-up period, the no diabetes group had a total of 97 cardiac-related deaths (weighted mortality rate: 0.9%), the GDM group had 4 cardiac-related deaths (weighted mortality rate: 0.4%, HR 1.48; 95% CI, 0.50–4.39), and the overt diabetes group had 82 cardiac-related deaths (weighted mortality rate: 4.8%, HR 2.37; 95% CI, 1.69–3.32).

### Sensitivity analysis

**Fig. 3:**
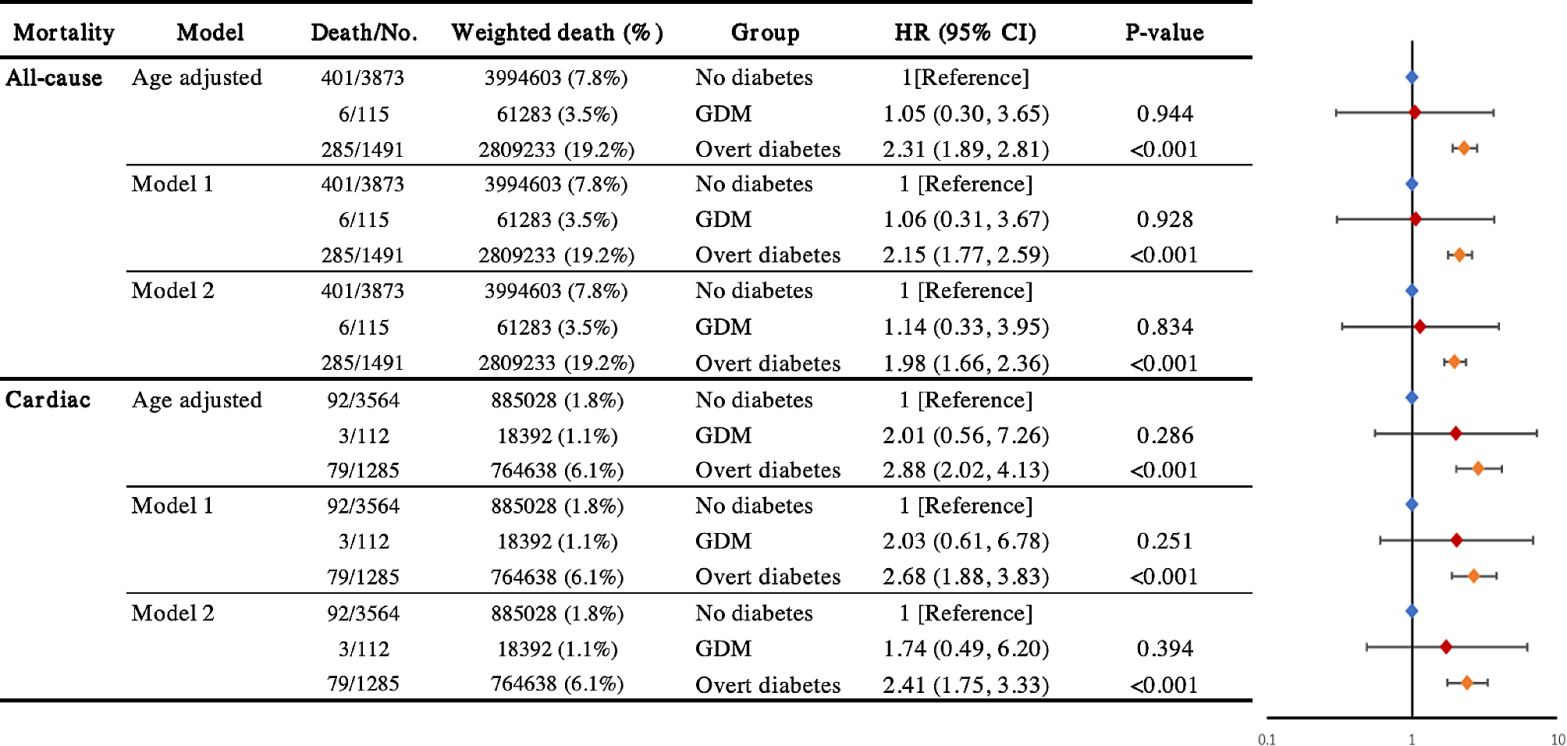
GDM With All-cause, Cardiac Mortality Among Us Women Aged 50 Years and Older With Pregnancy History, NHANES 2007 to 2018. Model 1 used multivariable model adjusted for age, race and ethnicity, education, family poverty income ratio, BMI, smoking status, alcohol consumption, and physical activity. Model 2 additionally adjusted for no. of pregnancies, hypertension, hypercholesterolemia, history of cardiovascular disease or cancer

For women aged over 50 years, as shown in Fig. 3, during the follow-up period, the no diabetes group had 401 deaths (weighted mortality rate: 7.8%), the GDM group had 6 deaths (weighted mortality rate: 3.5%, HR 1.14; 95% CI, 0.33–3.95), and the overt diabetes group had 285 deaths (weighted mortality rate: 19.2%, HR 1.98; 95% CI, 1.66–2.36). In terms of cardiac-related deaths during the follow-up period, the no diabetes group had a total of 92 deaths (weighted mortality rate: 1.8%), the GDM group had 3 deaths (weighted mortality rate: 1.1%, HR 1.74; 95% CI, 0.49–6.20), and the overt diabetes group had 79 deaths (weighted mortality rate: 6.1%, HR 2.41; 95% CI, 1.75–3.33).

## Discussion

In this study, we examined a representative cohort of 10,327 women (weighted population: 132,332,187) with pregnancy history in the United States. Approximately 5.3% of women had GDM alone, while about 14.1% had overt diabetes, and 80.6% had no diabetes. The follow-up period lasted up to 13.3 years, during which a total of 69,063 person-years of observation were recorded. Our findings indicated that there was no significant association between a history of GDM alone and increased risk of all-cause or cardiac mortality. However, we did observe a significant association between overt diabetes and increased risk of both all-cause and cardiac mortality. Similar results were observed in our analysis of women aged over 50 years.

Our findings suggest that there are different risks of cardiovascular death between GDM and overt diabetes, which may be attributed to the physiological effects of these conditions on the body. Overt diabetes is a complex metabolic disorder characterized by persistent hyperglycemia. Prolonged hyperglycemia can impair endothelial cells, promote the development of atherosclerosis and plaque formation, and ultimately lead to stenosis and obstruction of the coronary artery. Moreover, hyperglycemia can induce chronic inflammation and oxidative stress reactions, further exacerbating cell and vascular damage. At the same time, diabetes can contribute to myocardial injury and impaired contractile function. This increases the risk of a series of cardiovascular diseases, including coronary artery disease, cardiomyopathy, and heart failure, ultimately leading to an increased risk of cardiovascular death [13–17].

On the other hand, GDM occurs when maternal β-cell insulin secretion fails to compensate for the gradually increasing insulin resistance during pregnancy, and typically resolves after delivery [18]. GDM can have an impact on perinatal outcomes, including a higher incidence of preterm birth, neonatal hypoglycemia, macrosomia, maternal and neonatal birth injuries [19–23]. However, it has been increasingly recognized that women with a history of GDM may also experience long-term health issues, even if postpartum glucose levels return to normal. In the past few years, several studies have assessed the relationship between GDM and the occurrence of cardiovascular disease (CVD) later in life. These studies generally indicate that women with a history of GDM have a significantly increased risk of developing various CVD and metabolic disorders in the future [24–28]. A meta-analysis of five clinical studies including 390,591,101 women (424,101 CVD events) fount that GDM increases the risk of CVD events in the future, which is not entirely dependent on whether it progresses to diabetes. Even without developing diabetes, the risk of CVD still increases [29]. The precise mechanism behind this effect is still not fully understood, but a new concept has been proposed [30], suggesting that women with GDM already have a preexisting high-risk cardiac metabolic phenotype before pregnancy, which persists during and after pregnancy but is only detected through routine glucose tolerance screening during pregnancy. Prolonged exposure to cardiac metabolic abnormalities compared to their peers may play a role in the onset of GDM and the occurrence of CVD later in life. In addition, the differential risk of CVD between women with GDM and those without GDM was highest in the first decade after the index pregnancy and decreased over time [29]. This may explain our study results, that GDM increases the risk of CVD in young women (childbearing age) within 10 years after delivery, but the absolute incidence rate is still low, and this effect gradually weakens over time. Therefore, when considering the entire life cycle, a history of GDM does not have an influence on the risk of cardiac mortality.

Apart from its impact on cardiovascular system, overt diabetes can give rise to complications in multi organ system, including the kidneys, nervous system, and eyes [31]. Multiple studies have also indicated a potential association between GDM and various disease, including hepatic, renal, neoplastic, and psychiatric conditions [32–36]. These effects may be modulated by the subsequent development of diabetes but are not solely dependent on it. Unlike the various complications of diabetes that lead to a significant increase in all-cause mortality, we did not observe any significant association between a previous diagnosis of GDM and all-cause mortality. Like previously stated, the effects of hyperglycemia in overt diabetes are direct and persistent, whereas a history of GDM may represent more of a state of chronic metabolic abnormality.

In summary, the occurrence of GDM may be related to long-term health problems in women, but whether it affects their mortality remains unknown. Our study is the first prospective study on long-term mortality of GDM, and we found that a history of GDM alone is not associated with all-cause or cardiovascular mortality. For young women, our follow-up period may not have been long enough, but similar conclusions were drawn when analyzing women over 50 years old separately. Women with a history of GDM do not need to be overly concerned about their mortality risk, but should focus more on long-term metabolic management after delivery. We recommend that women with a history of GDM actively adjust their lifestyle, such as dietary adjustments and increased physical activity, and undergo long-term follow-up, with blood glucose testing as the main monitoring item, to timely detect whether they develop overt diabetes. In addition, patients with overt diabetes have significantly increased long-term mortality and cardiovascular mortality risks, and we recommend increasing monitoring item of these patients, including cardiac examinations, and actively controlling blood glucose. Having a better understanding of the long-term prognosis associated with GDM and overt diabetes can assist us in effectively distributing healthcare resources.

The strengths of our study include the use of a nationally representative sample, enhancing the generalizability of our results. Additionally, we adjusted for a range of potential confounding factors in our analysis. Nevertheless, it is important to acknowledge several limitations in our study. Firstly, the information on GDM was self-reported. Secondly, while we considered lifestyle as confounding factors in our analysis, these factors may change over time, and we were unable to capture the dynamic changes of these variables.

## Conclusions

In this prospective cohort study involving women with a pregnancy history in the United States, 5.3% of women had GDM alone. Our findings revealed that a history of GDM did not show any significant association with increased risks of overall mortality or cardiac mortality. However, women with overt diabetes exhibited a notable increase in mortality risk. Long-term health follow-up is necessary for women with a history of GDM.

## Data Availability

The datasets generated and analysed during the current study are available in the website: https://wwwn.cdc.gov/nchs/nhanes/continuousnhanes/default.aspx.
